# Infection of the Central Nervous System, Sepsis and Amyotrophic Lateral Sclerosis

**DOI:** 10.1371/journal.pone.0029749

**Published:** 2011-12-27

**Authors:** Fang Fang, Honglei Chen, Karin Wirdefeldt, Lars-Olof Ronnevi, Ammar Al-Chalabi, Tracy L. Peters, Freya Kamel, Weimin Ye

**Affiliations:** 1 Department of Medical Epidemiology and Biostatistics, Karolinska Institutet, Stockholm, Sweden; 2 Epidemiology Branch, National Institute of Environmental Health Sciences, Research Triangle Park, North Carolina, United States of America; 3 Department of Clinical Neuroscience, Karolinska Institutet, Stockholm, Sweden; 4 King's College London, MRC Centre for Neurodegeneration Research, Institute of Psychiatry, London, United Kingdom; University Hospital La Paz, Spain

## Abstract

**Background:**

Severe infections may lead to chronic inflammation in the central nervous system (CNS) which may in turn play a role in the etiopathogenesis of amyotrophic lateral sclerosis (ALS). The relentless progression and invasive supportive treatments of ALS may on the other hand induce severe infections among ALS patients.

**Methodology and Principal Findings:**

The present study included 4,004 ALS patients identified from the Swedish Patient Register during 1991–2007 and 20,020 age and sex matched general population controls. Conditional logistic regression was used to estimate the odds ratios (ORs) of ALS given a previous hospitalization for CNS infection or sepsis. Cox models were used to estimate the hazard ratios (HRs) of hospitalization for CNS infection or sepsis after ALS diagnosis. Overall, previous CNS infection (OR: 1.3, 95% confidence interval [CI]: 0.8, 2.4) or sepsis (OR: 1.2, 95% CI: 0.9, 1.6) was not associated with ALS risk. However, compared to ALS free individuals, ALS cases were more likely to be hospitalized for sepsis after diagnosis (HR: 2.6, 95% CI: 1.9, 3.5). We did not observe a higher risk of CNS infection after ALS diagnosis.

**Conclusions/Significance:**

Our results suggest that acute and severe infections unlikely contribute to the development of ALS; however, ALS patients are at a higher risk of sepsis after diagnosis, compared to ALS free individuals.

## Introduction

Amyotrophic lateral sclerosis (ALS) is a neurodegenerative disease mainly affecting motor neurons. About 5–10% of ALS patients have a familial origin and the rest are sporadic cases. Twin studies suggest a heritability of sporadic ALS of 0.61 with the unshared environmental component as 0.39 [1]. Genes found mutated in familial ALS have also been seen in sporadic ALS [2]. It is statistically possible that rare variants could account for the genetic component of apparently sporadic cases [3]. Environmental exposures remain important either by contributing to ALS risk independently or by interacting with genetic variations.

A chronic inflammatory process in the central nervous system (CNS) has repeatedly been suggested as a pathophysiological component in the disease [4] and the potential utility of anti-inflammatory therapy has attracted much research interest [5], [6]. A less well addressed question is however the origins of neuroinflammation. One possibility is mutant superoxide dismutase 1 (SOD1) which may activate microglia and damage neurons and thus induce a vicious cycle of inflammation in the CNS [7]. However, SOD1 mutations are present in only 20% of the familial ALS cases and are rare in sporadic cases, so other sources of neuroinflammation may also be important.

Acute immune responses to severe infections may also initiate sustained chronic inflammatory processes in the CNS. In animal experiments, both intracerebral and intraperitoneal challenges by lipopolysaccharide (LPS) induce sustained inflammations in the CNS and subsequent neuron death [8]. In addition, viral infections have long been suspected as potential contributors to ALS pathogenesis [9], [10]. For example, enteroviral components accompanied by the infiltration of peripheral immune cells have been found in the CNS of sporadic ALS cases [11], [12]. However, to the best of our knowledge, no epidemiological study has been conducted to evaluate the role of severe infections in relation to ALS risk. Using the Swedish Patient Register which has independently collected hospital records for infections and ALS, we tested the hypothesis that previous CNS infection or sepsis might increase the future risk of ALS.

Severe infections may also be a consequence of ALS, due to the deteriorating overall health condition of the patients and potentially also due to the use of invasive treatments. Via examination of death certificates, previous studies often showed that complications with respiratory infections at the terminal stages of ALS are common [13], [14], [15], [16], [17], [18]. However, there are little data on other infections after ALS diagnosis. We therefore further tested the hypothesis that ALS patients were more likely to be hospitalized for CNS infection or sepsis compared to individuals free of ALS.

## Materials and Methods

### Ethics Statement

The study was approved by the Regional Ethical Vetting Board in Stockholm, Sweden, who waived the need for consent as we only analyzed de-identified data.

### Swedish Patient Register

Since 1964–1965, the Swedish National Board of Health and Welfare has compiled data on individual hospital discharge diagnoses coded according to the *International Classification of Diseases* (ICD) (ICD-7 before 1969, ICD-8 for 1969–1986, ICD-9 for 1987–1996, and ICD-10 for 1997 and onward). This Inpatient Register (IPR) started in a few counties and became nationwide in 1987. The Outpatient Register (OPR) was established in 2001, covering outpatient visits to specialist care. Both the IPR and the OPR (referred to collectively as the Patient Register) record one primary diagnosis and up to eight secondary diagnoses for each clinic visit, along with information on the dates of contact and discharge.

**Figure 1:**
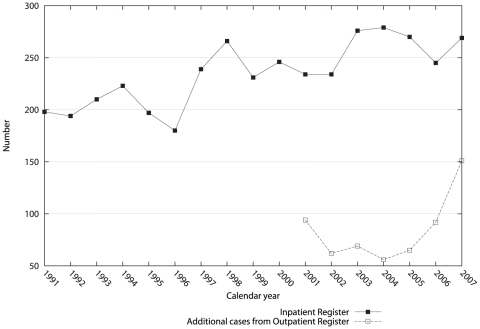
Number of amyotrophic lateral sclerosis patients identified in the Inpatient and Outpatient Registers in Sweden during 1991–2007.

We identified ALS cases from both the IPR and the OPR. **Figure 1** illustrates the annual number of the IPR cases during 1991–2007 and the additional cases identified from the OPR during 2001–2007, in the entire country. The majority of the ALS cases (87%) could be identified from the IPR. More additional cases were seen in 2001 and during 2006–2007 from the OPR, the former probably due to the inclusion of prevalent cases at the beginning of the OPR and the latter likely due to newly diagnosed cases not yet hospitalized.

### Nested case-control study – previous infections and ALS risk

We conducted a nested case-control study within the population of all individuals that were born in Sweden during 1901–1970 and included in the Swedish Population and Household Survey in 1990. The National Registration Number (NRN), a unique identifier for each resident in Sweden, enables unambiguous linkage across various health and population registers held by the Swedish National Board of Health and Welfare and the Statistics Sweden. Via the unique NRN, the whole study population was followed between January 1^st^ 1991 and December 31^st^ 2007 through linkages to the Swedish Patient, Causes of Death, and Emigration Registers.

Cases were defined as individuals with a first-ever identifiable inpatient or outpatient visit where ALS was recorded as either the primary or a secondary diagnosis in the Patient Register (1991–1996 ICD-9 code: 335C and 1997–2007 ICD-10 code: G12.2). The date of the first hospital visit was defined as the diagnosis date for ALS. Individuals that had died, emigrated out of Sweden or been hospitalized for ALS before January 1^st^ 1991 were excluded from the cohort. Follow-up was censored at ALS diagnosis, emigration out of Sweden, or death, whichever occurred first. A total of 4,004 ALS patients were identified during the study period, the majority (91%) with ALS as the primary diagnosis. Before 2001, cases could only be identified from the IPR (n = 1,938). Between 2001 and 2007, there were 2,066 cases including 393 cases (19%) that were identifiable only in the IPR, 483 (23%) only in the OPR, and 1,190 (58%) in both the IPR and the OPR. Among the latter cases, 390 (33%) were first recorded in the IPR and later in the OPR and 800 (67%) vice versa. The median interval between the first OPR record and the first IPR record for the 800 cases was 5.2 months. Assuming that the first OPR record serves as the real diagnosis date of ALS, the delay of the IPR identification is likely to be no more than a few months.

Using incidence density sampling, we randomly selected 20,020 controls (five per case) from the entire study population that were individually matched to the ALS cases on age and sex. Eligible controls were persons that were alive and free of ALS on the diagnosis date of the case they were matched to (i.e., index date). With this nested case-control design, we estimated a statistical power of >80% for detecting an odds ratio (OR) of 1.6, assuming 1% of infection among controls and an α of 0.05.

Our exposure of interest was hospitalization due to CNS infection or sepsis identified from the IPR. To ensure a complete identification of such hospitalizations, we defined the exposure status of the cases and controls between January 1^st^ 1987, when the IPR achieved nationwide coverage, and the index date. We used ICD codes for CNS infection and sepsis according to a previously published study which compared the records of infections in the Swedish Patient Register with medical records from the intensive care units [19]. Briefly, CNS infections were defined as ICD-9 codes 006F, 013, 036A, 036B, 045–049, 052B, 053A, 053B, 054D, 054H, 055A, 056A, 062–064, 072B, 072C, 094, 136C, and 320–325, and ICD-10 codes A06.6, A17, A39, A80–A89, B00.3, B00.4, B01.0, B01.1, B02.0, B02.1, B05.0, B05.1, B06.0, B22.0, B26.1, B26.2, B37.5, B38.4, B43.1, B50.0, B58.2, B60.2, G00–G08, and R29.1. For sepsis, we used the narrow criteria of sepsis as described in the same paper with ICD-9 codes 036C-036E, 036X, 038, 084, 112F, 117D, 286G, and 999D, and ICD-10 codes A02.1, A04.0-A04.3, A39–A41, A42.7, A48, A90–A99, B37.7, B38.7, B39.3, B40.7, B41.7, B42.7, B44.7, B45.7, B46.4, B95–B99, D65, and T80.2. The codes for CNS infections had a sensitivity of 84.0% and specificity of 99.7% while the codes for sepsis had a lower sensitivity (∼43%) but still a high specificity (∼97%) [19].

### Follow-up of ALS cases and controls – infections after ALS

To examine the relative risk of hospitalizations for CNS infection or sepsis after ALS diagnosis, we prospectively followed the cases and controls from the index date to death, emigration out of Sweden or December 31^st^ 2007, whichever came first.

### Statistical analysis

To evaluate the association of previous hospitalizations for CNS infection or sepsis with a later ALS occurrence, we derived ORs and corresponding 95% confidence intervals (CIs) from conditional logistic regression models. The exposure was first defined as an ever hospitalization for CNS infection or sepsis from January 1^st^ 1987 to the index date, and then categorized according to the time interval between such hospitalizations and ALS diagnosis as sample size allowed: <3 years and ≥3 years for CNS infection and <1 year, 1 to 3 years, and ≥3 years for sepsis. These analyses were conducted to explore the temporal relationship between exposures and outcome. In case of multiple hospitalizations, the first event was used in the primary analysis. Consecutive transfers among different clinics were counted as one hospitalization. We also examined the importance for ALS risk of number of hospitalizations (once or more than once) at least 3 years before the index date, assuming a 3-year induction time for ALS.

In addition to the main analysis we conducted parallel analyses restricted to cases first identified in the OPR, assuming that the first OPR record was the real date of diagnosis of ALS. Since some prevalent cases might have been included in the OPR during 2001 when it was started, we further restricted these analyses to individuals first included in the OPR in 2002–2007 (termed “incident cases”, n = 1,050).

The follow-up of ALS patients and controls after the index date identified no case but 35 controls that were hospitalized for CNS infection and therefore the analysis of ALS with later hospitalizations for infections was only conducted for sepsis. Hazard ratios (HRs) and 95% CIs were derived from Cox proportional hazards models. To illustrate the importance of the immediate period after diagnosis, we further stratified the analyses by time since index date, i.e., first year after index date and >1 year after index date.

All statistical analyses were performed with SAS 9.1 (SAS Institute, North Carolina, USA) and the significance tests were two-tailed with α = 0.05.

## Results

Incident cases were about 3 years younger on average (P<0.001) compared to other cases (Table 1). On average, cases with previous CNS infection were the same age as cases without previous CNS infection; whereas cases with previous sepsis were older than cases without previous sepsis (P = 0.006), especially if sepsis happened <3 years before ALS diagnosis (**Table 1**). Cases with sepsis before diagnosis were more likely to be male (P = 0.007; χ^2^ test).

**Table 1:** Basic characteristics of amyotrophic lateral sclerosis cases, a nested case-control study in Sweden, 1991–2007.

Groups	No.	Mean age at diagnosis(SD), years	Men (%)
All cases	4,004	68.8 (11.5)	2,242 (56.0)
Incident cases*	1,050	66.6 (11.5)	606 (57.7)
Other cases	2,954	69.5 (11.4)	1,636 (55.4)
Previous infection of the central nervous system			
No	3,989	68.8 (11.5)	2,232 (56.0)
Yes	15	68.7 (13.2)	10 (66.7)
<3 yrs before index date	5	72.2 (16.5)	4 (80.0)
≥3 yrs before index date	10	67.0 (11.9)	6 (60.0)
Previous sepsis			
No	3,935	68.7 (11.5)	2,192 (55.7)
Yes	69	72.6 (10.9)	50 (72.5)
<3 yrs before index date	43	74.3 (9.5)	30 (69.8)
≥3 yrs before index date	26	69.6 (12.6)	20 (76.9)

*Cases first recorded in the Outpatient Register during 2002–2007.

Overall, there was no association between ALS occurrence and previous hospitalization for either CNS infection (OR: 1.3, 95% CI: 0.8, 2.4) or sepsis (OR: 1.2, 95% CI: 0.9, 1.6) (**Table 2**). However, cases were more likely to be hospitalized for sepsis in the year preceding diagnosis compared to controls (OR: 2.6, 95% CI: 1.6, 4.2). This observation did not persist when the analysis was restricted to incident cases (OR: 1.0, 95% CI: 0.3, 2.9). Occurrence of multiple hospitalizations for either CNS infection or sepsis ≥3 years before the index date was not associated with a higher risk of ALS, but the estimates are based on small numbers (**Table 2**). To further examine the temporal relationship between infections and ALS, we evaluated separately associations of ALS with infections occurring ≥3 to <10 years previously and ≥10 years previously. ORs for the former period were 1.7 (95% CI: 0.8, 3.9) for CNS infection and 0.7 (95% CI: 0.5, 1.2) for sepsis; ORs for the latter period were 0.6 (95% CI: 0.1, 2.7) for CNS infection and 0.9 (95% CI: 0.4, 2.1) for sepsis.

**Table 2:** Previous hospitalizations for infection of the central nervous system (CNS) or sepsis and the risk of amyotrophic lateral sclerosis, a nested case-control study in Sweden, 1991–2007.

Groups	All cases and controls (N = 24,024)			Incident cases and controls (N = 6,300)†		
	Controls	Cases	OR (95% CI)*	Controls	Cases	OR (95% CI)*
Previous CNS infections						
No	19,964	3,989	1.0	5,230	1,048	1.0
Yes	56	15	1.3 (0.8–2.4)	20	2	0.5 (0.1–2.1)
Years before index date						
<3	17	5	1.5 (0.5–4.0)	4	0	-
≥3	39	10	1.3 (0.6–2.6)	16	2	0.6 (0.1–2.7)
No. of infections ≥3 years before index date						
1	34	9	1.3 (0.6–2.8)	13	2	0.8 (0.2–3.4)
>1	5	1	1.0 (0.1–8.6)	3	0	-
Previous sepsis						
No	19,728	3,935	1.0	5,145	1,035	1.0
Yes	292	69	1.2 (0.9–1.6)	105	15	0.7 (0.4–1.2)
Years before index date						
<1	52	27	2.6 (1.6–4.2)	20	4	1.0 (0.3–2.9)
1∼3	71	16	1.1 (0.7–2.0)	23	5	1.1 (0.4–2.8)
≥3	169	26	0.8 (0.5–1.2)	62	6	0.5 (0.2–1.1)
No. of infections ≥3 years before index date						
1	155	24	0.8 (0.5–1.2)	55	5	0.4 (0.2–1.1)
>1	14	2	0.7 (0.2–3.2)	7	1	0.7 (0.1–5.8)

*Odds ratios and 95% confidence intervals, adjusting for year of birth and sex.

† Cases first recorded in the Outpatient Register during 2002–2007.

Among cases, 71 (incidence rate: 11.4/1000 person-years) were hospitalized for sepsis at least once after the index date, with the first hospitalization for sepsis occurring on average 26 months later. The corresponding numbers for controls were 803 (incidence rate: 6.6/1000 person-years) and 65 months respectively. Overall, ALS cases were more likely than controls to be hospitalized for sepsis during the follow-up (HR: 2.6, 95% CI: 1.9, 3.5). During the first year after the index date, 85 controls and 29 cases were hospitalized for sepsis with an HR of 3.0 (95% CI: 1.9, 4.8) while comparing cases to controls (**Table 3**). Similar results were obtained after restricting the analyses to incident cases and their controls.

**Table 3:** Hazard ratios of hospitalization for sepsis after amyotrophic lateral sclerosis, a follow-up study in Sweden, 1991–2007.

Groups	All cases and controls (N = 24,024)			Incident cases and controls (N = 6,300)†		
	No. hospitalizedfor sepsis	Person-years	HR (95% CI)*	No. hospitalizedfor sepsis	Person-years	HR (95% CI)*
First year after index date						
Controls	85	19,046	1.0	24	4,740	1.0
Cases	29	2,541	3.0 (1.9–4.8)	10	812	3.0 (1.4–6.7)
>1 year after index date						
Controls	730	102,745	1.0	79	10,297	1.0
Cases	43	3,690	2.3 (1.6–3.4)	9	926	1.6 (0.8–3.5)

*Hazard ratios and 95% confidence intervals, adjusting for year of birth and sex.

† Cases first recorded in the Outpatient Register during 2002–2007.

## Discussion

This is the first large epidemiological study to examine the interrelationship of CNS infection or sepsis with ALS. We found little evidence that prior CNS infection or sepsis increases the future risk of ALS occurrence. However, our data suggest that ALS patients are at a higher risk of hospitalization for sepsis than individuals without ALS.

The strengths of the present study include the large number of ALS cases and controls, independently and prospectively collected data on infections and ALS, and the complete follow-up. However, even using these large national registers, many analyses were still based on small numbers due to the rarity of both the exposures and outcome. Another potential limitation is that ALS cases were identified from the Patient Register and, before 2001, solely from the IPR. It is therefore likely that we missed cases who had not been hospitalized either specifically for ALS or for other reasons. However, inclusion of patients from the OPR after 2001 should allow us to capture most of, if not all, cases since ALS patients often need to visit a specialist for a definitive diagnosis and treatment. Furthermore, the fact that IPR patients were hospitalized with a mean of 5 months after first OPR identification suggests that even the IPR might have identified ALS cases in a timely manner. As cases were identified from the registers, we did not have detailed data on ALS diagnosis and clinical features, such as the date and site of onset. Finally, although no specific validation has been conducted for ALS diagnosis in the Patient Register, clinical diagnosis of ALS is believed to be accurate and correlates well with post-mortem diagnosis [18] and the general quality of diagnoses in the IPR has been shown to be high [20]. We have observed a high agreement between the IPR and the OPR in the present study as well as between the IPR and the Causes of Death Register previously [21].

A disturbed homeostasis of microglia and a transformation of microglia from resting to reactivated status is a prominent feature of ALS pathology [6]. The initial function of microglia is to maintain the homeostatic balance of the microenvironment in the CNS, and this “surveillance” task is usually accomplished while facing mild insults. Strong insults to the CNS, such as infectious challenges or significant brain injuries, may incur excessive microglia activation and inflammatory responses. These inflammatory responses may persist after the acute insult, eventually turning into a self-perpetuating hazardous cycle, and thus contribute to neuronal damage [22], [23]. In the present study, we studied hospitalizations for sepsis or CNS infection as proxies for severe systemic and CNS infections that are more likely to have long-term neurological consequences than mild peripheral infections. Sepsis is a systemic inflammatory syndrome in response to infections. In contrast to our expectations, neither CNS infection nor sepsis was associated with higher ALS occurrence. The positive association observed between sepsis during the year before index date and ALS may most likely be due to reverse causality given that ALS patients appear more prone to sepsis than controls. In support of this suggestion, we did not observe a higher ALS risk associated with sepsis among incident cases, while we did find a higher sepsis risk among ALS cases after diagnosis.

One possible explanation for our finding of no association between severe infections and ALS risk may be that severe infections influence disease progression rather than initiation. In experimental models, intracerebral or intraperitoneal injection of LPS leads to an exacerbated neuroinflammation during chronic neurodegeneration [8], whereas intraparenchymal injection of LPS in the normal brain provokes only limited inflammatory response [24]. This suggests that the influences of such challenges might be more pronounced for ALS progression, when neurodegeneration is already ongoing, compared to ALS onset. This line of reasoning is consistent with the finding in a mouse model that selective mutation of SOD1 in motor neurons but not glia determines the onset of ALS while the mutation confined to microglial cells influences the progression of ALS after disease onset [25], [26]. Similarly, although few studies have evaluated the impact of infection on Alzheimer's disease risk, some epidemiological and clinical studies have shown that infections accelerate the progression of Alzheimer's disease [27], [28]. Therefore, future investigations of the association between severe infections and ALS survival, independent of other known prognostic indicators, are warranted.

Another potential explanation for our null finding is that the infections of the exposed individuals were likely well controlled after hospitalization, making the exposure a severe but transient event. Antibiotic treatment given to mice with systemic infections sufficiently controls neuroinflammation, seemingly explaining the null effect of recurrent systemic infectious challenges on ALS onset and progression in this model [29]. A transient infection, however severe, might not be enough to initiate neurodegeneration, whereas a sustained long term chronic infection might have a stronger disease modulating influence. Studies able to collect information on more chronic infections, such as septic arthritis, should be considered.

It is known that ALS patients often die of infectious complications in the respiratory system, possibly secondary to ventilatory failure caused by respiratory muscle weakness in the terminal stages of ALS [13], [14], [15], [16], [17], [18]. The overall deteriorating condition of ALS patients, such as malnutrition, in addition to respiratory failure, might put ALS patients at a higher risk of severe infections including sepsis. Furthermore, ALS patients may undergo significantly invasive treatments including tracheostomy or percutaneous endoscopic gastrostomy placement which could potentially also lead to an increased risk of sepsis. Despite the rareness of sepsis, our data suggest that it is a severe complication for ALS patients after diagnosis. This may have clinical implications for health professionals and caregivers of ALS patients. On the other hand, we observed no cases but 35 controls that were hospitalized for CNS infection after the index date, making analyses on CNS infection after ALS diagnosis impossible. This observation is however not surprising. Assuming a similar hospitalization rate of CNS infection between the cases and controls, the expected number of ALS cases hospitalized for CNS infection after diagnosis is only 1.8.

In conclusion, our study did not support a positive association between previous hospitalization for CNS infection or sepsis and an increased risk of ALS. Future studies should address whether these conditions are related to ALS prognosis. ALS patients are at a higher risk of sepsis after diagnosis and this should be taken into consideration in the clinical care of ALS.
